# COI metabarcoding primer choice affects richness and recovery of indicator taxa in freshwater systems

**DOI:** 10.1371/journal.pone.0220953

**Published:** 2019-09-12

**Authors:** Mehrdad Hajibabaei, Teresita M. Porter, Michael Wright, Josip Rudar

**Affiliations:** 1 Centre for Biodiversity Genomics at Biodiversity Institute of Ontario and Department of Integrative Biology, University of Guelph, Guelph, Ontario, Canada; 2 Natural Resources Canada, Great Lakes Forestry Centre, Sault Ste. Marie, Ontario, Canada; University of California San Diego, UNITED STATES

## Abstract

Mixed community or environmental DNA marker gene sequencing has become a commonly used technique for biodiversity analyses in freshwater systems. Many cytochrome c oxidase subunit I (COI) primer sets are now available for such work. The purpose of this study is to test whether COI primer choice affects the recovery of arthropod richness, beta diversity, and recovery of target assemblages in the benthos kick-net samples typically used in freshwater biomonitoring. We examine six commonly used COI primer sets on samples collected from six freshwater sites. Biodiversity analyses show that richness is sensitive to primer choice and the combined use of multiple COI amplicons recovers higher richness. Thus, to recover maximum richness, multiple primer sets should be used with COI metabarcoding. In ordination analyses based on community dissimilarity, samples consistently cluster by site regardless of amplicon choice or PCR replicate. Thus, for broadscale community analyses, overall beta diversity patterns are robust to COI marker choice. Recovery of traditional freshwater bioindicator assemblages such as Ephemeroptera, Trichoptera, Plectoptera, and Chironomidae as well as Arthropoda site indicators were differentially detected by each amplicon tested. This work will help future biodiversity and biomonitoring studies develop not just standardized, but optimized workflows that either maximize taxon-detection or the selection of amplicons for water quality or Arthropoda site indicators.

## Introduction

DNA-based biodiversity analysis has gained major attention due to the use of high throughput sequencing technology in approaches such as mixed community or environmental DNA metabarcoding [1,2]. Data generation typically involves DNA extraction from an environmental sample such as water or soil, or from collected biomass such as benthic kicknet or malaise trap followed by PCR amplification of one or more taxonomic markers such as the COI DNA barcode region and subsequent high throughput sequencing and bioinformatic analysis of marker gene sequences. Resulting sequences are then assigned to sequence clusters (Operational Taxonomic units, OTUs; Exact Sequence Variants, ESVs) and/or taxonomic names [3]. Sequence clusters and taxonomic lists obtained are used in various statistical analyses for assessing different aspects of biodiversity such as species richness or distribution, community composition, and functional diversity [4]. In practice, these questions are often geared towards identifying assemblages or specific target taxa. Biodiversity information gained can contribute to ecological investigations and applications such as biomonitoring as part of environmental assessment programs [5,6].

A major step in obtaining sequence data from mixed community or environmental samples involves PCR amplification of target marker gene(s). An important consideration in this multi-template PCR step is the choice of primer sets. It has been shown that primers can differentially bind to template DNA and this can result in both qualitative and quantitative biases [7–9]. Although a single COI primer set has been used to show congruence in metabarcoding and morphological biomonitoring methods [10], other studies have tested the performance of different COI amplicons on a phylogenetically diverse set of natural [11] and mock target taxa [12] to find growing evidence that multiple COI amplicons can provide better biodiversity coverage from environmental samples [13].

Indicators can be used to distinguish among conditions or sites. Traditionally, water quality indicators such as Ephemeroptera (mayflies), Plecoptera (stoneflies), and Trichoptera (caddisflies) are known to be sensitive to water pollution whereas Chironomidae (non-biting midges) have been shown to be tolerant to high levels of pollution [14,15] and we collectively refer to this assemblage as the EPTC. In the rapidly growing field of DNA-based biomonitoring, the detection of EPTC have also been used to guide primer development [16] and compare the performance of metabarcoding versus conventional methods for freshwater biomonitoring [10]. Useful indicators can also be identified from metabarcoding data to identify which sequences or taxa are significantly associated with sites using techniques originally developed for species indicator analysis [17]. Because of the difficulties associated with morphological identification of larval samples from benthos, samples are generally identified to family or genus level. Sorting and identifying individual samples from benthos poses a serious challenge in executing large-scale biomonitoring programs. With the advancement of genomics methods such as DNA metabarcoding, sequence data generated from whole communities can be used to provide biodiversity information on richness, beta diversity, community composition, and target taxa such as water quality and site indicators.

The objective of this study was to test the performance of several newly published COI metabarcode primers to detect freshwater benthic invertebrates. We wanted to determine the impact of primer choice on several components of diversity: richness, beta diversity, and recovery of bioindicators. We tested a total of six partial COI metabarcode amplicons, including the BR5 and F230R amplicons that we have used routinely for macroinvertebrate monitoring [13,18,10,19].

## Methods

### Field methods

Six benthic invertebrate communities were sampled from shallow streams across the City of Waterloo (Ontario, Canada) using a modified travelling kick-and-sweep technique outlined in the Ontario Benthos Biomonitoring Network protocol [20] (S1 Table, S1 Fig). Briefly, wetted width was measured and used to calculate the number of return trips required to sample a 10 m transect of the stream specifically targeting a riffle habitat. Prior to sampling D-nets were decontaminated by soaking them in a 10% bleach solution for 15 min, rinsing with tapwater, and drying them overnight. A clean 500 μm mesh D-net was held downstream to the person sampling, with the opening of the net facing the person sampling. Substrate was disturbed by kicking the substrate at a constant effort for 3 minutes across the 10 m transect dislodging invertebrates and allowing the flowing water to guide the dislodged macroinvertebrates into the net. The entire contents inside the net including substrate, with the exception of rocks and twigs (which were rinsed with 100% ethanol before removal), were transferred from the net to a clean 1 L polyethylene bottle, preserved with 80% ethanol and stored at -20°C until further processing in the lab. No specific permits is required to sample freshwater benthos using kicknet in our sampling locations and we did not sample any endangered species.

### Molecular biology methods

#### DNA extraction

Samples were homogenized separately in a clean blender (decontaminated thoroughly with Eliminase solution (Decon Labs: King of Prussia, PA, USA) (Black and Decker Model: BL2010BGC), distributing 50 mL of the homogenate into six sterile conical tubes, one for each sample. Samples were centrifuged at 2400 x g for 2 min to collect homogenate at the bottom of the tube, and excess preservative ethanol was removed. Samples were covered and incubated at 65°C until residual ethanol was evaporated (roughly 5–8 hours). DNA was extracted using Qiagen’s DNeasy PowerSoil kit (Toronto, Canada. Product Ref: 12888) according to the manufacturer’s protocol, eluting with 30 μL molecular biology grade water. All samples were extracted concurrently with the inclusion of one negative control for the batch where no sample was added.

#### Polymerase chain reaction

**Fig 1:**
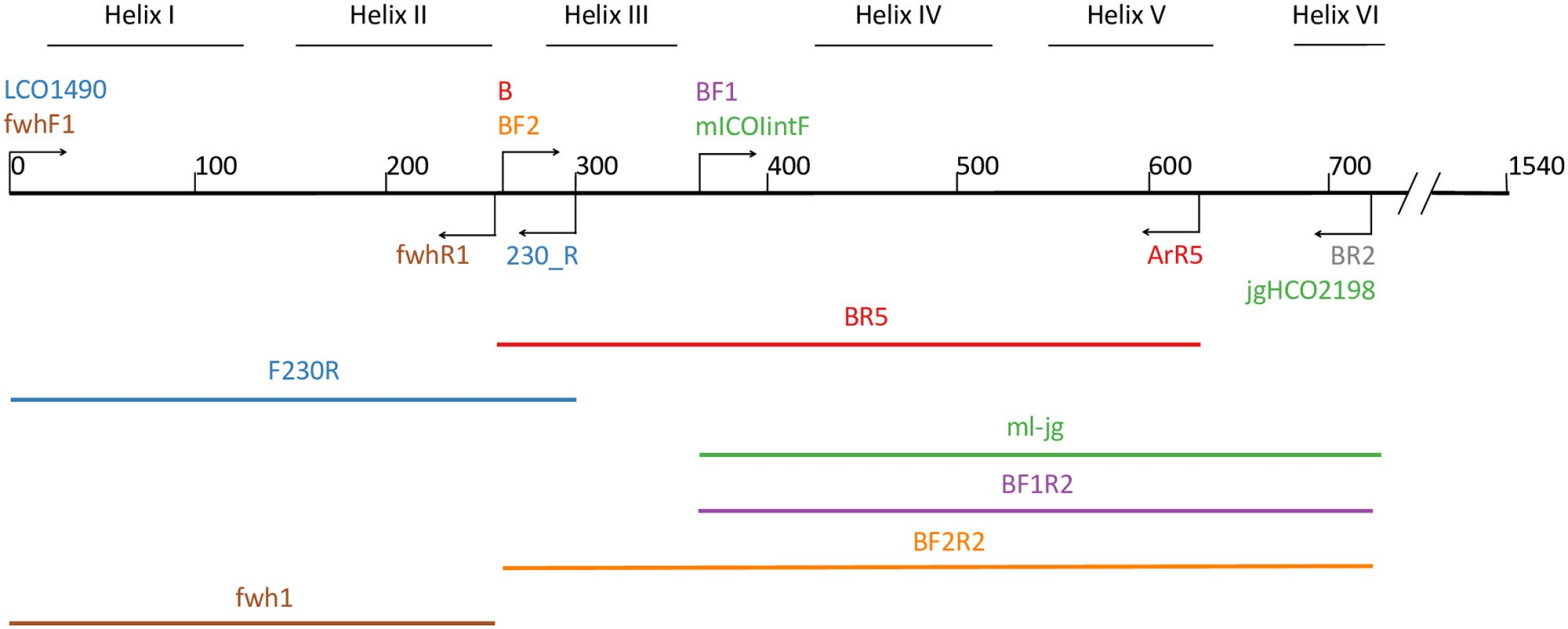
Map of primers and amplicons tested in this study. The reference sequence shown in black is *Drosophila yakuba*, cytochrome c oxidase region 1470–3009 bp (1540 nt). Secondary structure is shown for reference, comprised of six alpha helices in the standard DNA barcode region shown here.

The six amplicons from COI barcode region used in this study are shown in Fig 1. The primers were aligned against the *Drosophila yakuba* COI barcode region to match the sequence used to design the original Folmer primers obtained from GenBank accession X03240 using Mesquite v3.10 [21]. Since well-defined COI secondary structure is not available for *D*. *yakuba* or any other insect that we are aware of, we used structural information from *Bos taurus* from UniProt accession P00396. All samples were amplified for six primer sets according to their published amplification regime (Table 1) with the exception that a two-step PCR was used for all reactions (first PCR using untailed primers, second PCR using Illumina adapter-tailed primers), even if a one-step PCR was used in the original protocol. PCRs were run in duplicate with a negative control. Amplification success was confirmed through gel electrophoresis (not pictured). Amplicons were purified using a MinElute PCR Purification kit, quantified on a TBS-380 Mini-Fluorometer (Turner Biosystems Sunnyvale California, United States) using a Quant-iT PicoGreen dsDNA assay (Invitrogen Waltham Massachusetts, United States Product Ref: P11496). The concentration of each purified amplicon was normalized individually and two amplicons were pooled for each sample and tagged. This resulted in three pairs of tagged pooled amplicons per sample and this was done because the run contained samples from other experiments and we wanted to ensure equal sequencing coverage. Tags were added in a third PCR of 12 cycles to add Illumina’s Nextera Indexes (San Diego, California, United States Product Ref: FC-121-1011) which allow for samples to be multiplexed in the same run. All indexed samples were pooled, purified through magnetic bead purification, quantified using the PicoGreen dsDNA assay, and average fragment length for the library was determined on an Agilent Bioanalyzer 2100 (Santa Clara, California, United States. Product ref: G2939BA) using the Agilent DNA 7500 assay chip (Product Ref: 5067–4627). The library was diluted then sequenced using Illumina’s MiSeq v3 sequencing chemistry kit (2x300 cycle. Product Ref: MS-102-3003) on an Illumina MiSeq, comprising approximately half of a sequencing run. The sequencing run included a 10% PhiX spike-in as a control and to add more sequence heterogeneity to the plate.

**Table 1:** COI amplicons used in this study.

COI Amplicon	Primer	Target group	5’-3’ Primer sequence	Mode amplicon length (bp)	Primer reference	PCR conditions
BR5	B	Freshwater benthic macroinvertebrates	CCIGAYATRGCITTYCCICG	310	[22]	95°C for 5min, 35 cycles of 94°C for 40s, 46°C for 1min, and 72°C for 30s, and a final extension at 72°C for 5min
	ArR5*	Tropical Arthropods	GTRATIGCICCIGCIARIACIGG		[13]	
F230R	LCO1490	Metazoan invertebrates	GGTCAACAAATCATAAAGATATTGG	229	[23]	95°C for 5min, 35 cycles of 94°C for 40s, 46°C for 1min, and 72°C for 30s, and a final extension at 72°C for 5min
	230_R	Arthropods	CTTATRTTRTTTATICGIGGRAAIGC		[18]	
ml-jg	mlCOIintF	Metazoa	GGWACWGGWTGAACWGTWTAYCCYCC	313	[24]	95°C for 1 min, 35 cycles of 94°C for 15 s, 46°C for 15 s, 72°C for 10s, and final extension at 72°C for 3 min
	jgHCO2198	Marine invertebrates	TAIACYTCIGGRTGICCRAARAAYCA		[25]	
BF1R2	BF1	Freshwater macroinvertebrates	ACWGGWTGRACWGTNTAYCC	316	[16]	94 °C for 3 min; 40 cycles of 94 °C for 30 s, 50 °C for 30 s, and 65 °C for 2 min; and final extension at 65 °C for 5 min
	BR2	Freshwater macroinvertebrates	TCDGGRTGNCCRAARAAYCA			
BF2R2	BF2	Freshwater macroinvertebrates	GCHCCHGAYATRGCHTTYCC	421	[16]	94 °C for 3 min; 40 cycles of 94 °C for 30 s, 50 °C for 30 s, and 65 °C for 2 min; and final extension at 65 °C for 5 min
	BR2	Freshwater macroinvertebrates	TCDGGRTGNCCRAARAAYCA			
fwh1	fwhF1	Freshwater macroinvertebrates	YTCHACWAAYCAYAARGAYATYGG	178	[26]	95°C for 5 min, 34 cycles of 95°C for 30 s, 52°C for 30 s, 72°C for 2 min, and final extension at 72°C for 10 min
	fwhR1	Freshwater macroinvertebrates	ARTCARTTWCCRAAHCCHCC			

### Bioinformatic processing

Raw sequences were processed with the SCVUC COI metabarcode pipeline v2.1 available from GitHub at https://github.com/Hajibabaei-Lab/SCVUC_COI_metabarcode_pipeline. The acronym SCVUC stands for the major programs or algorithms used for bioinformatic processing: “S”–SEQPREP, “C”–CUTADAPT, “V”–VSEARCH, “U”–UNOISE, “C”–COI Classifier. Briefly, this semi-automated pipeline is described below. Jobs were spread across multiple cores using GNU Parallel [27]. Raw compressed fastq Illumina read files were paired using SeqPrep specifying a minimum Phred score of 20 at the ends of the reads and an overlap of at least 25 bp [28]. The following steps were conducted separately for each of the six amplicons tested in this study. Primers were trimmed using CUTADAPT v1.14 and reads were retained if they were at least 150 bp long after trimming, had a minimum Phred score of 20 at the ends of the reads, and contained no more than 3 N’s. CUTADAPT was also used to convert fastq files to FASTA files [29]. The individual sample files were combined into a single file for global ESV generation. VSEARCH v2.4.2 was used to dereplicate the data (get the unique reads) using the–derep_fulllength option [30]. The USEARCH v10.0.240 unoise3 algorithm was used to denoise the reads [31]. This involved the removal of any contaminating PhiX reads (carry over from Illumina sequencing), prediction and removal of sequences with errors, removal of putative chimeric sequences, and removal of rare sequences. We defined rare sequences to be those clusters comprised of less than 3 reads (singletons and doubletons) [32,33]. We used this set of exact sequence variants (ESVs) as a reference, and all primer trimmed reads were mapped to this reference set with an identity of 1.0 (100% sequence similarity) to generate a sample x ESV table. The COI Classifier v3.2, that uses a naïve Bayesian classifier v2.12 with a custom COI reference set, was used to taxonomically assign the ESVs [34]. This naïve Bayesian classifier, popular in the microbial ecology community, was trained on a COI reference dataset mined from GenBank to address a gap in existing COI bioinformatic tools [35,36]. This method allowed us to quickly process large batches of COI metabarcodes generated from high throughput sequencing to provide a measure of statistical confidence for each taxonomic assignment, at each rank, instead of just reporting a measure of sequence similarity. This method has been shown to be faster and have a lower false positive rate than the top BLAST hit method [35,36]. Briefly, the COI classifier we use here breaks down each query sequence into a series of 8 bp words or k-mers, uses k-mer frequencies and a naïve Bayesian approach to make a taxonomic assignment, then calculates the statistical confidence for the assignment at each rank from species to superkingdom. Taxonomic assignments were mapped to ESVs detected in each sample with a custom Perl script. The final taxonomy table for each primer was concatenated.

### Data analysis

The final taxonomy table was formatted in R v3.4.3 in RStudio v1.1.419 [37,38]. Custom scripts are available from GitHub at https://github.com/Hajibabaei-Lab/HajibabaeiEtAl2019. Data was summarized multiple taxonomic ranks. High confidence taxonomic assignments were retained by filtering for bootstrap support cutoffs > = 0.30 at the genus rank and > = 0.20 at the family rank. Using these cutoffs ensures that 95–99% of the taxonomic assignments are correct, assuming our query taxa are in the reference database [36]. We retained taxa at the species rank with a bootstrap support cutoff > = 0.70. Assuming our query species are present in the reference database, this should ensure that at least 95% of species level assignments are correct. To check whether we had sufficient sequencing depth, we used the package VEGAN v2.5–2 to plot rarefaction curves using the ‘rarecurve’ function [39]. Curves that reach a plateau show saturated sequencing. To account for variable library sizes, reads/library were rarefied down to the 15^th^ percentile library size using the ‘rrarefy’ function [40].

We compared richness recovered from each amplicon, from each site, using the VEGAN ‘specnum’ function and total richness was plotted with ggplot2 [41]. Richness data was checked for normality using visual distribution plots (ggdensity and ggqqplot, not shown) as well as using the Shapiro-Wilk test of normality (W = 0.97, p = 0.36) and this data was treated as normally distributed in comparisons [42]. We compared average site richness from each amplicon using paired t-tests with the Holm adjustment for multiple comparisons.

There is uncertainty in how to interpret read abundance from arthropod metabarcoding studies due to unexpected template to product ratios after PCR due to stochasticity and GC content [8] as well as the effect of primer bias and body size variation across life stages and species that can vary by orders of magnitude and affect recovery [43]. As a result, we chose to transform read abundance into presence-absence data for all subsequent analyses. We checked for Pearson correlations in the presence-absence of ESVs recovered from two PCR replicates using the ‘psych’ and ‘corrplot’ functions in R [44,45]. We corrected for multiple comparisons using the Holm adjustment method [46].

Indicator species can be used as a proxy to indicate differences among conditions or sites [17]. For example, in freshwater systems, the diversity of EPTC taxa have been used as water quality indicators [10]. In this study, we determined a set of arthropod site indicators using the INDICSPECIES package in R and the ‘multipatt’ function with default settings [17]. This function accepts a presence-absence matrix (ESVs or taxa x samples) and grouping data (for each site) and determines the ESVs or taxa that are preferentially associated with each group. The analysis was run separately for each amplicon and significant site indicators were selected if the resulting p-value was < = 0.05. We tested an array of COI amplicons for their ability to recover arthropod site indicators and the usual EPTC water quality indicators.

To test whether sample clusters are affected by COI amplicon choice or PCR replicate, we used non-metric multidimensional scaling. Plots were created using the vegan ‘metaMDS’ function using the default settings with two dimensions (scree plot not shown) and dissimilarities were calculated using Sorensen dissimilarities by selecting the method ‘bray’ and binary = TRUE then plotted with ggplot. Goodness of fit was calculated using the VEGAN ‘goodness’ function. To check whether we had homogenous dispersion of dissimilarities, an assumption of permutational multivariate analysis of variance (PERMANOVA), we created a dissimilarity matrix with the VEGAN ‘vegdist’ function, then calculated beta dispersion using the ‘betadisper’ function in R. We tested for significant heterogeneity using analysis of variance (ANOVA) in R. We checked for significant interactions among sites, amplicons, and replicates as well as the significance of group clusters with PERMANOVA using the VEGAN ‘adonis’ function with 999 permutations.

## Results

A total of 9,980,584 x 2 paired-end reads were generated for this study and they have been deposited to the NCBI SRA #PRJNA545426 (S2 Table). After pairing and primer trimming we retained a total of 7,619,108 reads. A summary of ESV counts for all taxa are shown in S3 Table. About 23% of raw reads were retained in the denoised set of ESVs whereas the difference was removed during denoising as putative sequence errors, chimeras, PhiX contamination, or rare singletons and doubletons. In this study, we tested an array of primers that are popular in the literature and/or newly developed even though some were designed to target arthropods specifically, or metazoan invertebrates more broadly (Table 1). For this reason, we limited our comparisons to arthropods which are expected to be amplified by all the primer sets. As shown in S2 Fig, the greatest number of reads were recovered from Arthropoda, but many reads were also recovered from Annelida, Proteobacteria, and other non-target phyla. About 24% of all ESVs were taxonomically assigned to Arthropoda taxa and the final Arthropoda ESV counts are shown in Table 2. When six COI primer pairs are compared, F230R ESVs contained the highest proportion of Arthropoda ESVs (43.9%) and contained the highest proportion of raw reads mapped to ESVs (4.7%). About 13% of raw reads were mapped to this final set of Arthropoda ESVs. Out of all the Arthropoda taxonomic assignments, 11% of unique species, 15% of genera, and 26% of families were considered high confidence assignments (S4 Table). The proportion of raw reads represented in these high confidence Arthropoda assignments was 7% for species, 8% for genera, and 10% for families.

**Table 2:** Arthropoda ESV and read counts vary by COI amplicon.

	BR5	F230R	ml-jg	BF1R2	BF2R2	fwh1	Total
Arthropoda ESVs	873	1,143	1,342	803	477	302	4,940
Proportion of all ESVs assigned to Arthropoda (%)^1^	25	43.9	40.6	13.1	13.7	15.5	23.5
Reads in Arthropoda ESVs	187,353	467,910	285,933	147,697	24,375	167,129	1,280,397
Proportion of raw reads in Arthropoda ESVs (%)^2^	1.9	4.7	2.9	1.5	0.2	1.7	12.8

^1^Number of Arthropoda ESVs from this table divided by the number of all ESVs from S3 Table multiplied by 100.

^2^Number of reads in Arthropoda ESVs from this table divided by the number of all reads in ESVs from S3 Table multiplied by 100.

Rarefaction curves show that at each rank, all samples reached a plateau, indicating that we had sufficient sequencing coverage for these samples (S3 Fig). The median Arthropoda richness was not significantly different across the pairwise amplicon comparisons (pairwise t-test, p > 0.05) (S4 Fig). The total number of unique Arthropoda taxa were compared across COI amplicons (S5 Fig) and the amplicon that detects the most unique taxa varied depending on the taxonomic resolution of the results. At the ESV rank, the ml-jg amplicon recovered the highest richness. We also note that the presence-absence of ESVs are positively correlated across 2 PCR replicates (S6 Fig).

**Fig 2:**
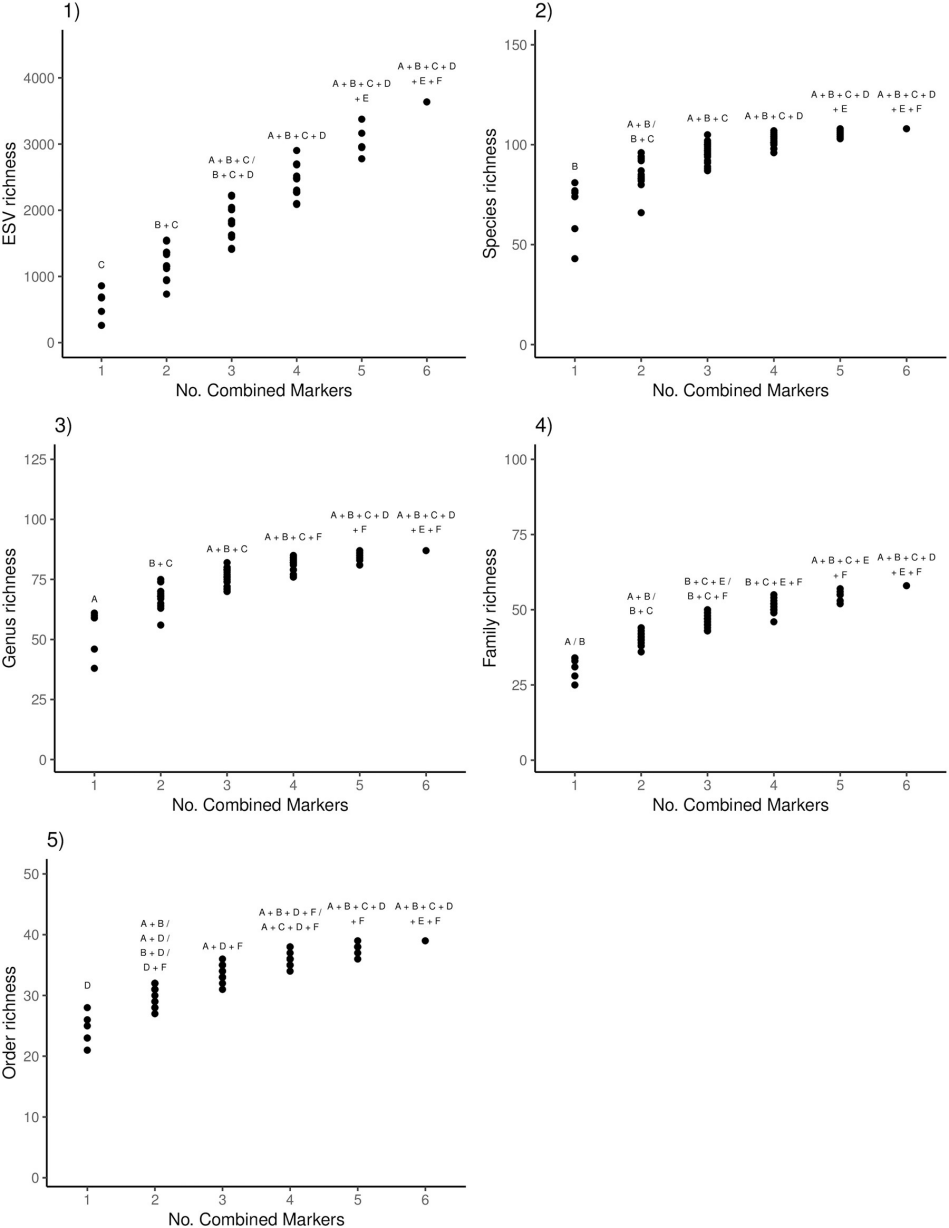
ESV richness continues to increase as COI amplicons are added but species—Order richness reaches a plateau. For the primer comparison experiment that used the soil DNA extraction kit, we pooled the results from the six sites and show the top COI amplicon combinations that detected the greatest richness. We report the recovered richness when up to six amplicons are combined at the 1) ESV, 2) species, 3) genus, 4) family, and 5) order ranks. ESV = exact sequence variant; A = BR5; B = F230R; C = ml-jg; D = BF1R2; E = BF2R2; F = fwh1.

To test the effect of using multiple COI amplicons on richness, we pooled increasing numbers of combined amplicons. We show that using a multi-amplicon approach can detect greater richness than using any single amplicon alone (Fig 2). In this study, ESV richness increases linearly as amplicons are added. Note that while richness is sensitive to the presence of artefactual sequence variants and that changing filtering and denoising parameters will likely affect absolute richness, the trend of increasing richness detected with additional sampled amplicons has been previously demonstrated and is likely due to the known effect of primer bias [13]. In some cases, multiple combinations of amplicons recover equivalent richness. Though the accumulation curves at the species, genus, and family ranks begin to plateau after sampling 2 amplicons, only the data points plotted at the ESV rank represents all the Arthropoda we detected. Note that only ~ 11%, 15%, and 26% of ESVs could be confidently identified to the species, genus, and family ranks, respectively, so these particular plots only represent a small slice of the diversity that we could confidently identify (S4 Table). Due to limitations in the underlying reference sequence databases [47], it is likely that species richness will also increase as additional reference taxa are added so that more ESVs can be assigned with high-confidence [48].

**Fig 3:**
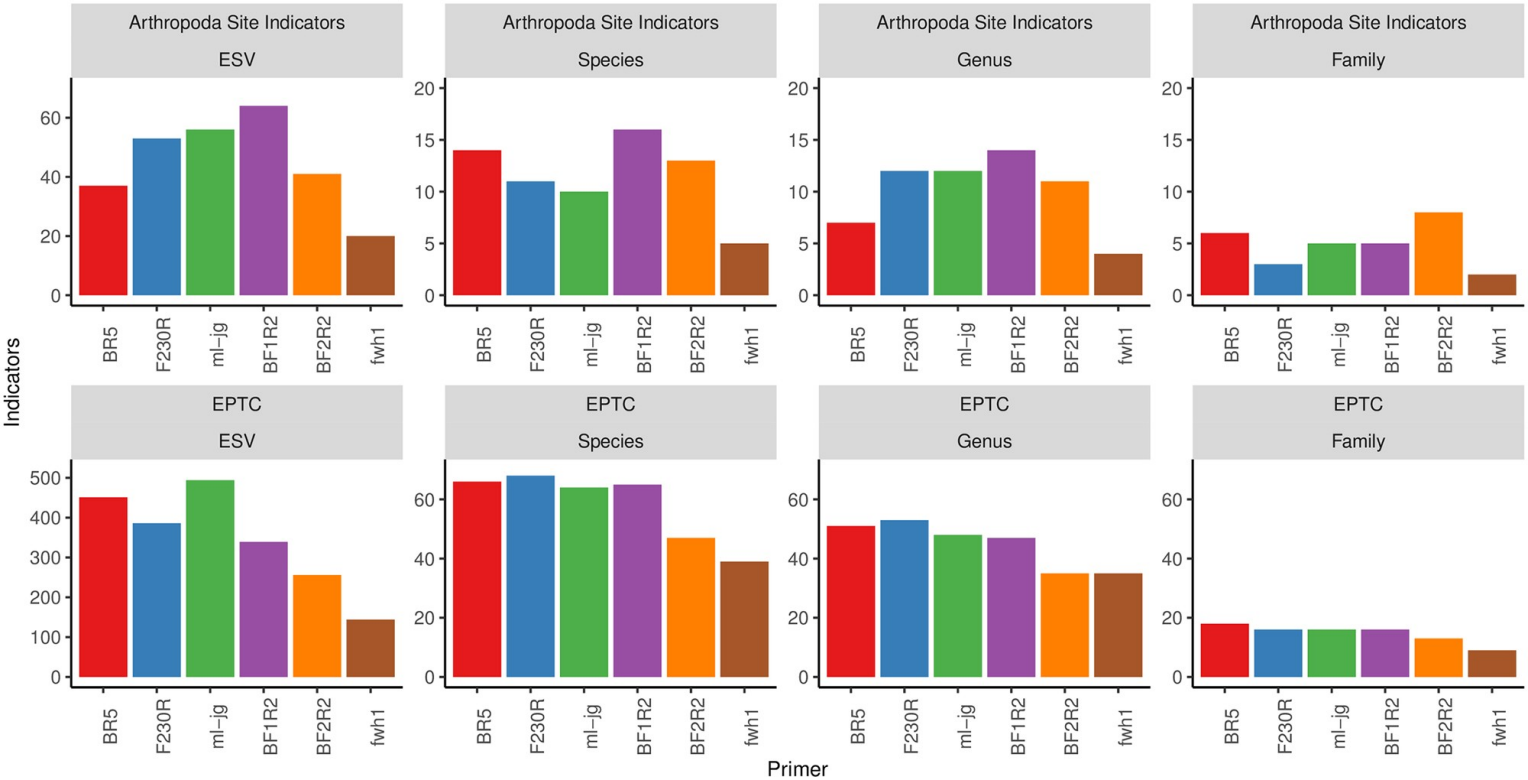
Each amplicon differentially recovers site and water quality indicators. In the top panel, the number of site indicator taxa from across the Arthropoda are shown. In the bottom panel, the number of typical water quality indicator taxa from the EPTC are shown. This analysis was based on normalized data. ESV = exact sequence variant; EPTC = Ephemeroptera, Plecoptera, Trichoptera, Chironomidae.

**Fig 4:**
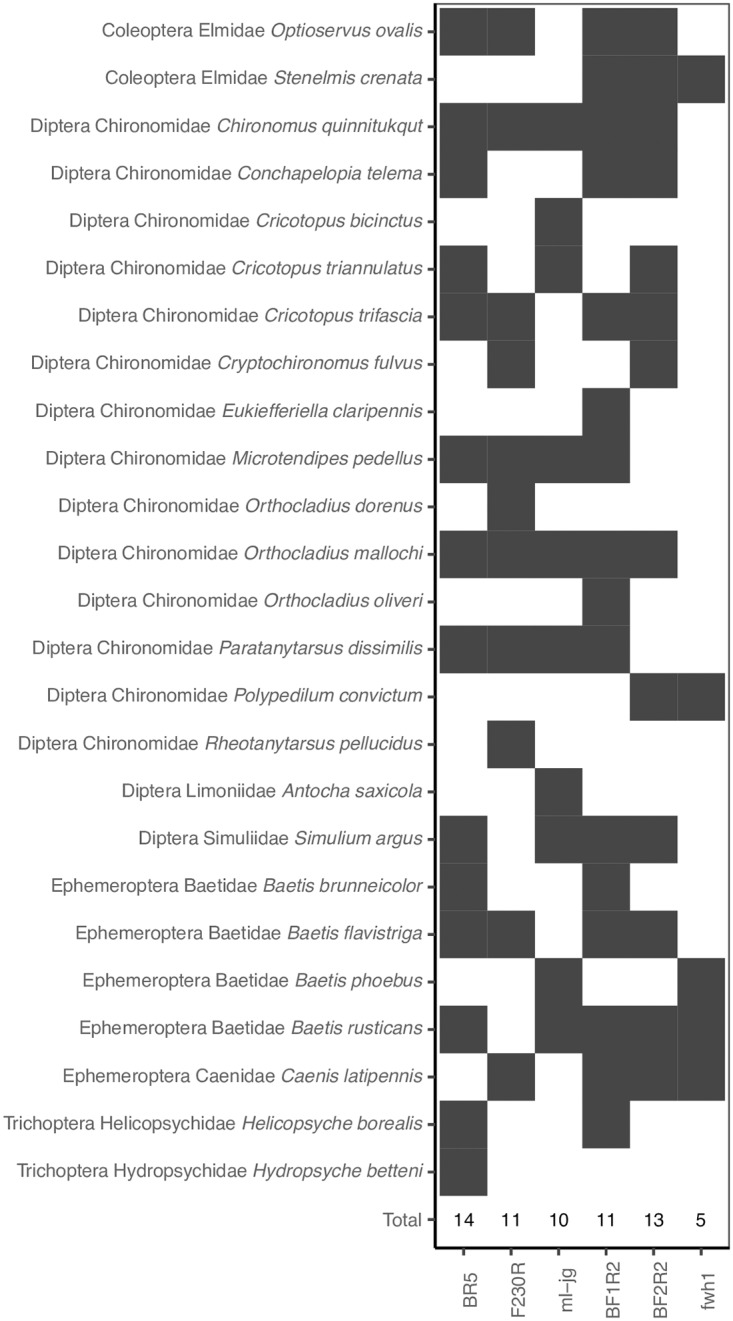
Site indicator taxa chosen based on metabarcode sequencing are comprised of Coleoptera, Diptera, Ephemeroptera, and Trichoptera. Presence is indicated by a dark square, absence by a white square. The total number of Arthropoda site indicator taxa detected by each amplicon is shown in the bottom row.

We also looked at how the recovery of Arthropoda site indicator taxa and water quality indicator taxa from the EPTC varied with amplicon choice (Fig 3). Generally, the amplicon that recovers the greatest number of site indicators varies according to the taxonomic resolution of the analysis. At the ESV rank, BF1R2 recovers the greatest number of arthropoda site indicator taxa and ml-jg specifically recovers the greatest number of EPTC. Since the subset of indicator taxa presented for the species to family ranks only represents the portion of the ESVs assigned with high confidence, rank specific results may change over time as reference databases better represent local taxa [48]. We plotted the taxonomic distribution of the Arthropoda site indicator species and how this varied for each amplicon (Fig 4). Site indicator taxa include Elmidae (riffle beetles), Limoniidae (crane flies), Simuliidae (black flies), Ephemeroptera, and Trichoptera.

**Fig 5:**
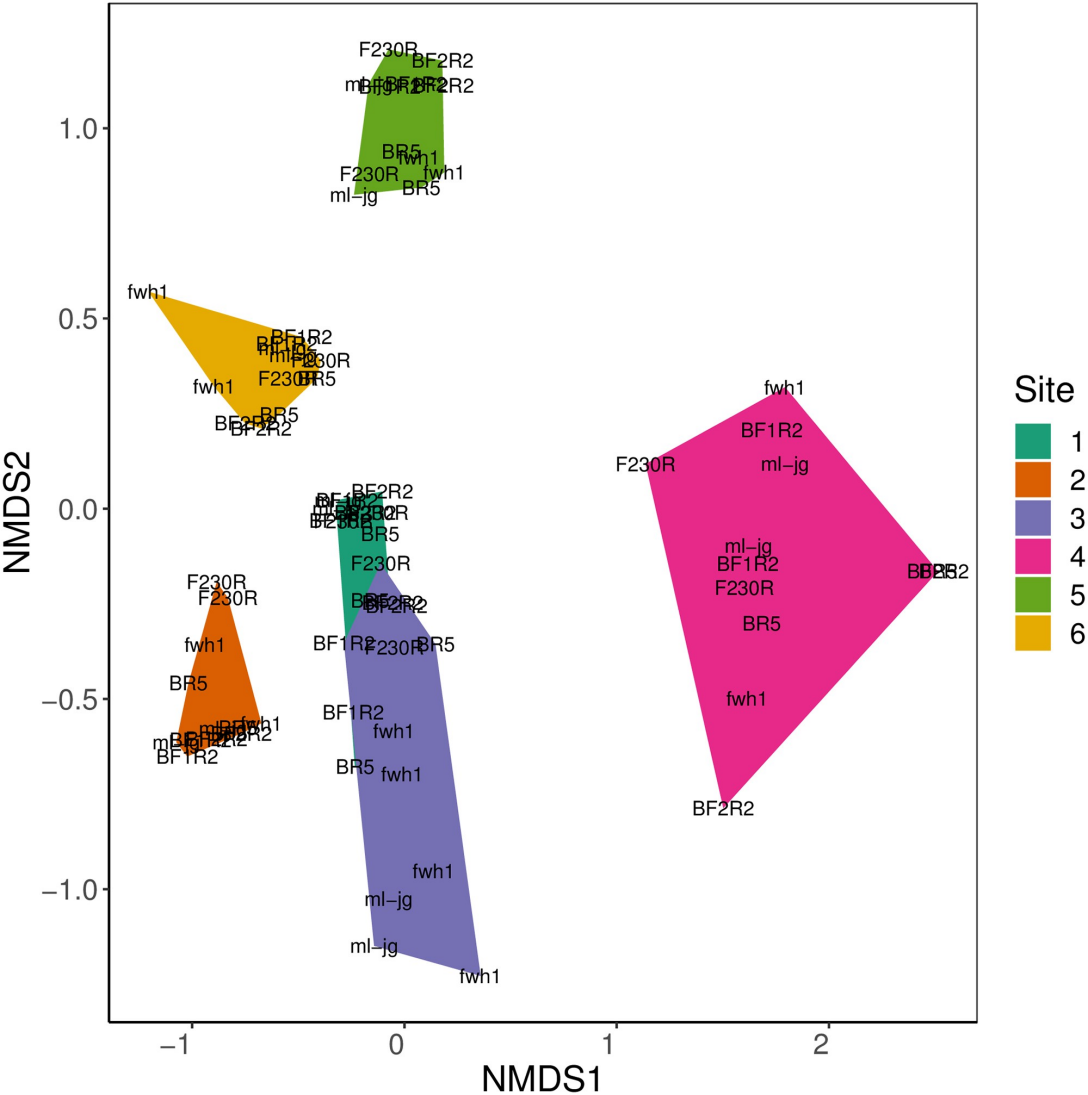
Samples cluster mainly by site despite differences in amplicons and replicates. Results are based normalized data. COI amplicons are labelled directly in the plot. Amplicons shown twice represent the two PCR replicates. Sites are grouped by color according to the legend.

To investigate the effect of amplicon choice on beta diversity we looked at how sites cluster with respect to COI amplicon choice and PCR replicates. We compared all the data at the ESV rank (Fig 5). Samples cluster by site (stress = 0.154, linear R^2^ = 0.912). We found significant heterogeneity of beta diversity among sites (p-value < 0.05), but since we had a balanced design, proceeded to use PERMANOVA to test the significance of groupings [49]. There were no significant interactions among sites, amplicons, or PCR replicates. Amplicon choice and PCR replicate did not explain any significant variation in beta diversity among samples, but sites explained 76% of the variation among samples (R^2^ = 0.76, p-value = 0.001).

## Discussion

As showcased in recent literature, DNA metabarcoding has gained significant popularity in various ecological studies where biodiversity in a habitat or a sample is investigated [50,51,13,18,52–54,4,55]. In this study we show that the optimal choice of amplicon(s) ought to be based on the objective of the study: optimizing richness, optimizing the differentiation of samples based on sites/conditions, or optimizing the detection of target taxa. Here we show the impact of using varied primer sets all of which have been used in recent metabarcoding studies of freshwater benthic macroinvertebrates.

As predicted, different primer sets produced varied richness results. For example, while the ml-jg amplicon produced the highest overall ESV richness, combinations of amplicons together detected even greater richness. Moreover, even though ml-jg maximizes ESV richness, at the species rank the best choice is F230R, yet at the genus rank BR5 optimizes richness. The decision to present the results of a study at various taxonomic ranks is often based on the desire to include all the data (ESV rank), or to present results at a fine level of taxonomic resolution (species rank), or to present results based on previous knowledge. For example, 94% of North American freshwater specimens identified by morphology are represented by a DNA sequence so it may be desirable to present results at the genus rank [47]. These observations have important implications for choosing primers, especially when considering the level of standardization required in biomonitoring programs. While the use of a single primer set is desirable to keep costs to a minimum, the trade-off is that only a subset of the total richness will be detected, especially in environments that comprise a phylogenetically diverse array of species. Based on results from this study and elsewhere, primer binding biases during amplification steps can have tangible impacts on results and using multiple primer sets will aid in increasing taxonomic coverage [56,57,22,13]. For the sake of flexibility and forward compatibility, aside from the deposition of raw data in public databases, we also encourage authors to provide denoised ESVs. As discussed in the literature, denoised ESVs represent the finest level of resolution for sequence-based biodiversity data and have a clear biological meaning as observed DNA sequences [3,58]. This is in contrast to operational taxonomic units (OTUs) that represent clouds of similar sequences with a single chosen representative sequence. When reports are summarized to other taxonomic ranks, we encourage disclaimer statements that results are limited by the taxonomic coverage of current reference databases that may improve in the future [48].

Our study provides important insights with regards to use of varied PCR primer sets and replicates. Contrary to measures of alpha diversity (above), beta diversity measures do not seem to be affected by primer sets or PCR replicates when ESVs are used for the spatial analysis. In other words, spatial separation of sites based on these varied parameters are robust as used in biomonitoring applications. These results are in line with previous studies that found alpha diversity, but not beta diversity, is sensitive to primer choice [59,60]. Indeed, any metric that is sensitive to the presence of rare species such as richness or indicator species analysis is unlikely to be robust [61]. For example, it has been shown that even with matched sequencing depth, the Illumina NovaSeq with patterned flow cells recovers greater richness than the MiSeq, making direct comparisons across studies using different sequencing technologies difficult [62]. The implication here is that beta diversity is less sensitive to primer choice and technical replicates so would be easier to compare across studies.

An important and widespread use of metabarcoding data is in determining ecosystem status or “biomonitoring” where the state of the ecosystem is derived from bioindicator assemblages such as EPTC [14]. The recovery of freshwater bioindicators from metabarcoding data in this study varied with amplicon choice. For example, F230R amplicon detects the greatest number of traditional EPTC site indicators. Metabarcoding data is also commonly used to distinguish among sites to test hypotheses about drivers of compositional differences. In this study, we compiled a list of arthropod site indicators as well as included all arthropod ESVs in ordination analyses to test compositional differences. We found that the recovery of arthropod site indicators varied with amplicon choice. For example, the BF1R2 amplicon detected the greatest number of arthropod site indicators. With the application of DNA-based methods, our ability to detect a broad range of taxa has improved such that it may not be necessary to limit sampling and reporting to traditional bioindicators since indicator assemblages can be parsed from whole community metabarcoding datasets as needed [55].

Note that even though equimolar amounts of each amplicon were combined for sequencing, variable numbers of reads were obtained across amplicons. This may be caused by variable amplification efficiency during library preparation or slight differences in the number of transferred amplicons when they are pooled prior to library preparation. Since the recovery of variable library sizes is such a common occurrence, it is important to normalize library size across samples prior to conducting data analysis. It has been shown that there is a trade-off between the use of rarefaction (removal of sequences such that each sample can be compared at a common library size) to reduce the false positive rate, and a loss of sensitivity because of the removal of sequences [63,64]. This has implications for beta diversity analyses, where false positives can occur when samples cluster by sequencing depth obscuring real differences, especially for samples with very small library sizes. Common normalization methods include rarefaction down to the smallest library size and working with proportions (ESV reads per sample / total reads per sample). A simulation study showed that rarefaction combined with the analysis of presence-absence data worked best to cluster samples when groups are substantially different [40]. For differential abundance testing, however, methods that take into consideration the compositional nature of metabarcode datasets (log ratio transformation) may be more appropriate [65,40,64].

## Conclusions

This study analyzed how arthropod richness, beta diversity, and recovery of site indicator taxa vary with COI amplicon choice. We show how richness is sensitive to primer choice and the combined use of multiple COI amplicons. Beta diversity is robust to primer choice and PCR replicates. We also note that some amplicons recover more Arthropoda site indicators or freshwater EPTC bioindicators than others, and this should be taken into consideration during experimental design to address the need to distinguish among field sites or assess water quality.

## Supporting information

S1 Table Collection sites. (DOCX)

S2 Table Reads counts for all taxa. (DOCX)

S3 Table ESV counts for all taxa. (DOCX)

S4 Table Filtering for high confidence Arthropoda identifications affects the proportion of assignments retained across taxonomic ranks. (DOCX)

S1 Fig Collection sites. The map shows where collection sites were located. We used the ggmap library in R with the ‘get_stamenmap’ function to create the map (Kahle and Wickham, 2013 The R Journal 5 (1): 144–161), then used the ggsn library to add a scale bar (Santos Baquero, 2019 https://CRAN.R-project.org/package=ggsn).
(PDF)

S2 Fig Taxonomic distribution of reads in ESVs. Number of ESVs and reads in ESVs are shown for: a) all taxa at the phylum rank, and b) Arthropoda focusing on the Ephemeroptera, Plectoptera, Trichoptera, and Chironomidae. Results summarize the denoised ESVs across all samples, before rarefaction.
(PDF)

S3 Fig Sequencing depth is saturated for most samples. For each primer, color coded as in the legend, there are 12 lines for the six field sampling sites x 2 PCR replicates. The vertical line indicates the 15^th^ percentile library size that was used to normalize variable library sizes for subsequent diversity analyses. ESVs = exact sequence variants.
(PDF)

S4 Fig Most primers recovered similar median Arthropoda richness across sites. The first panel shows the six COI markers tested. Based on normalized data. Results shown are for 2 pooled PCR replicates at the ESV rank. ESV = exact sequence variant.
(PDF)

S5 Fig The primer that detects the highest Arthropoda richness varies by rank. Richness from each COI marker at a variety of taxonomic ranks are shown. Results are based on normalized data. ESV = exact sequence variant.
(PDF)

S6 Fig ESV presence-absence is positively correlated between the first and second PCR replicate. Circle color and size reflect Pearson correlations. Only significant correlations with a p-value < = 0.05 are shown. Results are based on normalized data. ESV = exact sequence variants. Label naming convention is as follows: DNA extraction kit _ marker _ site _ PCR replicate. A = BR5, B = F230R, C = ml-jg, D = BF1, E = BF2, F = fwh1.
(PDF)

## References

[1] Hajibabaei M, Shokralla S, Zhou X, Singer GAC, Baird DJ. Environmental Barcoding: A Next-Generation Sequencing Approach for Biomonitoring Applications Using River Benthos. PLOS ONE. 2011;6: e17497 10.1371/journal.pone.0017497 21533287 PMC3076369

[2] Taberlet P, Coissac E, Pompanon F, Brochmann C, Willerslev E. Towards next-generation biodiversity assessment using DNA metabarcoding. Molecular ecology. 2012;21: 2045–2050. 10.1111/j.1365-294X.2012.05470.x 22486824

[3] Callahan BJ, McMurdie PJ, Holmes SP. Exact sequence variants should replace operational taxonomic units in marker-gene data analysis. The ISME Journal. 2017;11: 2639–2643. 10.1038/ismej.2017.119 28731476 PMC5702726

[4] Porter TM, Hajibabaei M. Scaling up: A guide to high-throughput genomic approaches for biodiversity analysis. Molecular Ecology. 2018;27: 313–338. 10.1111/mec.14478 29292539

[5] Baird DJ, Hajibabaei M. Biomonitoring 2.0: a new paradigm in ecosystem assessment made possible by next-generation DNA sequencing. Molecular ecology. 2012;21: 2039–2044. 22590728 10.1111/j.1365-294x.2012.05519.x

[6] Leese F, Bouchez A, Abarenkov K, Altermatt F, Borja Á, Bruce K, et al Why We Need Sustainable Networks Bridging Countries, Disciplines, Cultures and Generations for Aquatic Biomonitoring 2.0: A Perspective Derived From the DNAqua-Net COST Action. Advances in Ecological Research. Elsevier; 2018 pp. 63–99. 10.1016/bs.aecr.2018.01.001

[7] Suzuki MT, Giovannoni SJ. Bias caused by template annealing in the amplification of mixtures of 16S rRNA genes by PCR. Applied and environmental microbiology. 1996;62: 625–630. 8593063 10.1128/aem.62.2.625-630.1996 PMC167828

[8] Polz MF, Cavanaugh CM. Bias in template-to-product ratios in multitemplate PCR. Applied and environmental Microbiology. 1998;64: 3724–3730. 9758791 10.1128/aem.64.10.3724-3730.1998 PMC106531

[9] Clarke LJ, Soubrier J, Weyrich LS, Cooper A. Environmental metabarcodes for insects: *in silico* PCR reveals potential for taxonomic bias. Molecular Ecology Resources. 2014;14: 1160–1170. 10.1111/1755-0998.12265 24751203

[10] Emilson CE, Thompson DG, Venier LA, Porter TM, Swystun T, Chartrand D, et al DNA metabarcoding and morphological macroinvertebrate metrics reveal the same changes in boreal watersheds across an environmental gradient. Scientific Reports. 2017;7 10.1038/s41598-017-13157-x 28986575 PMC5630640

[11] Lobo J, Shokralla S, Costa MH, Hajibabaei M, Costa FO. DNA metabarcoding for high-throughput monitoring of estuarine macrobenthic communities. Scientific Reports. 2017;7 10.1038/s41598-017-15823-6 29142319 PMC5688171

[12] Braukmann TW, Ivanova NV, Prosser SW, Elbrecht V, Steinke D, Ratnasingham S, et al. Revealing the Complexities of Metabarcoding with a Diverse Arthropod Mock Community. 2018; 10.1111/1755-0998.13008 PMC6850013 30779309

[13] Gibson J, Shokralla S, Porter TM, King I, van Konynenburg S, Janzen DH, et al Simultaneous assessment of the macrobiome and microbiome in a bulk sample of tropical arthropods through DNA metasystematics. PNAS. 2014;111: 8007–8012. 10.1073/pnas.1406468111 24808136 PMC4050544

[14] Buss DF, Baptista DF, Silveira MP, Nessimian JL, Dorvillé LF. Influence of water chemistry and environmental degradation on macroinvertebrate assemblages in a river basin in south-east Brazil. Hydrobiologia. 2002;481: 125–136.

[15] Bonada N, Prat N, Resh VH, Statzner B. DEVELOPMENTS IN AQUATIC INSECT BIOMONITORING: A Comparative Analysis of Recent Approaches. Annual Review of Entomology. 2006;51: 495–523. 10.1146/annurev.ento.51.110104.151124 16332221

[16] Elbrecht V, Leese F. Validation and Development of COI Metabarcoding Primers for Freshwater Macroinvertebrate Bioassessment. Frontiers in Environmental Science. 2017;5: 11 10.3389/fenvs.2017.00011

[17] De Cáceres M, Legendre P. Associations between species and groups of sites: indices and statistical inference. Ecology. 2009;90: 3566–3574. 10.1890/08-1823.1 20120823

[18] Gibson J, Shokralla S, Curry C, Baird DJ, Monk WA, King I, et al Large-Scale Biomonitoring of Remote and Threatened Ecosystems via High-Throughput Sequencing. PLOS ONE. 2015;10: e0138432 10.1371/journal.pone.0138432 26488407 PMC4619546

[19] Erdozain M, Thompson DG, Porter TM, Kidd KA, Kreutzweiser DP, Sibley PK, et al Metabarcoding of storage ethanol vs. conventional morphometric identification in relation to the use of stream macroinvertebrates as ecological indicators in forest management. Ecological Indicators. 2019;101: 173–184. 10.1016/j.ecolind.2019.01.014

[20] Jones C, Somers KM, Craig B, Reynoldson TB. Ontario Benthos Biomonitoring Network: Protocol Manual. Toronto: Queens Printer for Ontario; 2007.

[21] Maddison WP, Maddison DR. Mesquite [Internet]. 2015. http://mesquiteproject.org

[22] Hajibabaei M, Spall JL, Shokralla S, van Konynenburg S. Assessing biodiversity of a freshwater benthic macroinvertebrate community through non-destructive environmental barcoding of DNA from preservative ethanol. BMC Ecology. 2012;12: 28 10.1186/1472-6785-12-28 23259585 PMC3542036

[23] Folmer O, Black M, Hoeh W, Lutz R, Vrijenhoek R. DNA primers for amplification of mitochondrial cytochrome c oxidase subunit I from diverse metazoan invertebrates. Molecular marine biology and biotechnology. 1994;3: 294–299. 7881515

[24] Leray M, Yang JY, Meyer CP, Mills SC, Agudelo N, Ranwez V, et al A new versatile primer set targeting a short fragment of the mitochondrial COI region for metabarcoding metazoan diversity: application for characterizing coral reef fish gut contents. Frontiers in Zoology. 2013;10: 34 10.1186/1742-9994-10-34 23767809 PMC3686579

[25] Geller J, Meyer C, Parker M, Hawk H. Redesign of PCR primers for mitochondrial cytochrome c oxidase subunit I for marine invertebrates and application in all-taxa biotic surveys. Mol Ecol Resour. 2013;13: 851–861. 10.1111/1755-0998.12138 23848937

[26] Vamos E, Elbrecht V, Leese F. Short COI markers for freshwater macroinvertebrate metabarcoding. Metabarcoding and Metagenomics. 2017;1: e14625 10.3897/mbmg.1.14625

[27] Tange O. GNU Parallel—The Command-Line Power Tool.; login: The USENIX Magazine. 2011;February: 42–47.

[28] St. John J. SeqPrep [Internet]. Downloaded 2016. https://github.com/jstjohn/SeqPrep/releases

[29] Martin M. Cutadapt removes adapter sequences from high-throughput sequencing reads. EMBnet journal. 2011;17: pp–10.

[30] Rognes T, Flouri T, Nichols B, Quince C, Mahé F. VSEARCH: a versatile open source tool for metagenomics. PeerJ. 2016;4: e2584 10.7717/peerj.2584 27781170 PMC5075697

[31] Edgar RC. UNOISE2: improved error-correction for Illumina 16S and ITS amplicon sequencing. bioRxiv. 2016;

[32] Brown SP, Veach AM, Rigdon-Huss AR, Grond K, Lickteig SK, Lothamer K, et al Scraping the bottom of the barrel: are rare high throughput sequences artifacts? Fungal Ecology. 2015;13: 221–225. 10.1016/j.funeco.2014.08.006

[33] Tedersoo L, Nilsson RH, Abarenkov K, Jairus T, Sadam A, Saar I, et al 454 Pyrosequencing and Sanger sequencing of tropical mycorrhizal fungi provide similar results but reveal substantial methodological biases. New Phytologist. 2010;188: 291–301. 10.1111/j.1469-8137.2010.03373.x 20636324

[34] Wang Q, Garrity GM, Tiedje JM, Cole JR. Naive Bayesian Classifier for Rapid Assignment of rRNA Sequences into the New Bacterial Taxonomy. Applied and Environmental Microbiology. 2007;73: 5261–5267. 10.1128/AEM.00062-07 17586664 PMC1950982

[35] Porter TM, Gibson JF, Shokralla S, Baird DJ, Golding GB, Hajibabaei M. Rapid and accurate taxonomic classification of insect (class Insecta) cytochrome c oxidase subunit 1 (COI) DNA barcode sequences using a naïve Bayesian classifier. Mol Ecol Resour. 2014;14: 929–942. 10.1111/1755-0998.12240

[36] Porter TM, Hajibabaei M. Automated high throughput animal CO1 metabarcode classification. Scientific Reports. 2018;8: 4226 10.1038/s41598-018-22505-4 29523803 PMC5844909

[37] RStudio Team. RStudio: Integrated Development for R [Internet]. 2016. http://www.rstudio.com/

[38] R Core Team. R: A Language and Environment for Statistical Computing [Internet]. 2017. https://www.R-project.org/

[39] Oksanen J, Blanchet GF, Friendly M, Kindt R, Legendre P, McGlinn D, et al. vegan: Community Ecology Package. R package version 2.5–2. [Internet]. 2018. https://CRAN.R-project.org/package=vegan

[40] Weiss S, Xu ZZ, Peddada S, Amir A, Bittinger K, Gonzalez A, et al Normalization and microbial differential abundance strategies depend upon data characteristics. Microbiome. 2017;5: 27 10.1186/s40168-017-0237-y 28253908 PMC5335496

[41] Wickham H. ggplot2: Elegant Graphics for Data Analysis [Internet]. New York: Springer-Verlag; 2009 http://ggplot2.org

[42] Shapiro SS, Wilk MB. An Analysis of Variance Test for Normality (Complete Samples). Biometrika. 1965;52: 591–611.

[43] Elbrecht V, Leese F. Can DNA-Based Ecosystem Assessments Quantify Species Abundance? Testing Primer Bias and Biomass—Sequence Relationships with an Innovative Metabarcoding Protocol. PLOS ONE. 2015;10: e0130324 10.1371/journal.pone.0130324 26154168 PMC4496048

[44] Wei T, Simko V. R package “corrplot”: Visualization of a Correlation Matrix (Version 0.84). [Internet]. 2017. https://github.com/taiyun/corrplot

[45] Revelle W. psych: Procedures for Psychological, Psychometric, and Personality Research [Internet]. 2018. https://CRAN.R-project.org/package=psych

[46] Holm S. A Simple Sequentially Rejective Multiple Test Procedure. Scand J Statist. 1979;6: 65–70.

[47] Curry CJ, Gibson JF, Shokralla S, Hajibabaei M, Baird DJ. Identifying North American freshwater invertebrates using DNA barcodes: are existing COI sequence libraries fit for purpose? Freshwater Science. 2018;37: 178–189. 10.1086/696613

[48] Porter TM, Hajibabaei M. Over 2.5 million COI sequences in GenBank and growing. PLoS ONE. 2018;13: e0200177 10.1371/journal.pone.0200177 30192752 PMC6128447

[49] Anderson MJ, Walsh DCI. PERMANOVA, ANOSIM, and the Mantel test in the face of heterogeneous dispersions: What null hypothesis are you testing? Ecological Monographs. 2013;83: 557–574. 10.1890/12-2010.1

[50] Bik HM, Porazinska DL, Creer S, Caporaso JG, Knight R, Thomas WK. Sequencing our way towards understanding global eukaryotic biodiversity. Trends in Ecology & Evolution. 2012;27: 233–243. 10.1016/j.tree.2011.11.010 22244672 PMC3311718

[51] Yu DW, Ji Y, Emerson BC, Wang X, Ye C, Yang C, et al Biodiversity soup: metabarcoding of arthropods for rapid biodiversity assessment and biomonitoring: Biodiversity soup. Methods in Ecology and Evolution. 2012;3: 613–623.

[52] Creer S, Deiner K, Frey S, Porazinska D, Taberlet P, Thomas WK, et al The ecologist’s field guide to sequence-based identification of biodiversity. Methods in Ecology and Evolution. 2016;7: 1008–1018. 10.1111/2041-210X.12574

[53] Leese F, Altermatt F, Bouchez A, Ekrem T, Hering D, Meissner K, et al DNAqua-Net: Developing new genetic tools for bioassessment and monitoring of aquatic ecosystems in Europe. Research Ideas and Outcomes. 2016;2: e11321 10.3897/rio.2.e11321

[54] Bush A, Sollmann R, Wilting A, Bohmann K, Cole B, Balzter H, et al Connecting Earth observation to high-throughput biodiversity data. Nature Ecology & Evolution. 2017;1: 0176 10.1038/s41559-017-0176 28812589

[55] Bush A, Compson Z, Monk W, Porter TM, Steeves R, Emilson E, et al. Studying ecosystems with DNA metabarcoding: lessons from aquatic biomonitoring. bioRxiv. 2019;

[56] Clarke J, Wu H-C, Jayasinghe L, Patel A, Reid S, Bayley H. Continuous base identification for single-molecule nanopore DNA sequencing. Nat Nano. 2009;4: 265–270. 10.1038/nnano.2009.12 19350039

[57] Bellemain E, Carlsen T, Brochmann C, Coissac E, Taberlet P, Kauserud H avard. ITS as an environmental DNA barcode for fungi: an in silico approach reveals potential PCR biases. BMC microbiology. 2010;10: 189 10.1186/1471-2180-10-189 20618939 PMC2909996

[58] Glassman SI, Martiny JB. Ecological patterns are robust to use of exact sequence variants versus operational taxonomic units. mSphere. 2018;3: e00148–18. 10.1128/mSphere.00148-18 30021874 PMC6052340

[59] Drummond AJ, Newcomb RD, Buckley TR, Xie D, Dopheide A, Potter BC, et al Evaluating a multigene environmental DNA approach for biodiversity assessment. GigaSci. 2015;4: 46 10.1186/s13742-015-0086-1 26445670 PMC4595072

[60] Grey EK, Bernatchez L, Cassey P, Deiner K, Deveney M, Howland KL, et al Effects of sampling effort on biodiversity patterns estimated from environmental DNA metabarcoding surveys. Scientific Reports. 2018;8 10.1038/s41598-018-27048-2 29891968 PMC5995838

[61] Haegeman B, Hamelin J, Moriarty J, Neal P, Dushoff J, Weitz JS. Robust estimation of microbial diversity in theory and in practice. ISME J. 2013;7: 1092–1101. 10.1038/ismej.2013.10 23407313 PMC3660670

[62] Singer GAC, Fahner NA, Barnes JG, McCarthy A, Hajibabaei M. Comprehensive biodiversity analysis via ultra-deep patterned flow cell technology: a case study of eDNA metabarcoding seawater. Sci Rep. 2019;9: 5991 10.1038/s41598-019-42455-9 30979963 PMC6461652

[63] McMurdie PJ, Holmes S. Waste Not, Want Not: Why Rarefying Microbiome Data Is Inadmissible. PLOS Comput Biol. 2014;10: e1003531 10.1371/journal.pcbi.1003531 24699258 PMC3974642

[64] Weiss SJ, Xu Z, Amir A, Peddada S, Bittinger K, Gonzalez A, et al Effects of library size variance, sparsity, and compositionality on the analysis of microbiome data. 10.7287/peerj.preprints.1157v1

[65] Gloor GB, Macklaim JM, Pawlowsky-Glahn V, Egozcue JJ. Microbiome Datasets Are Compositional: And This Is Not Optional. Frontiers in Microbiology. 2017;8 10.3389/fmicb.2017.02224 29187837 PMC5695134

