# Supplementary material for: COI metabarcoding primer choice affects richness and recovery of indicator taxa in freshwater systems
Source: PLoS One. 2019 Sep 12;14(9):e0220953. doi: 10.1371/journal.pone.0220953 (PMC6742397; doi:10.1371/journal.pone.0220953)
Supplement: S1 Table — (DOCX) [file pone.0220953.s001.docx]

**Table S1: Collection sites**

| **Sample** | **Region** | **Collected** | **Lat** | **Long** |
| --- | --- | --- | --- | --- |
| 1 | Laurel | May 31, 2018 | 43°28'2.64"N | 80°31'59.33"W |
| 2 | Clair | May 29, 2018 | 43°27'46.93"N | 80°32'56.75"W |
| 3 | Laurel | May 29, 2018 | 43°28'39.31"N | 80°33'30.45"W |
| 4 | Beaver | May 15 2018 | 43°29'28.24"N | 80°37'27.80"W |
| 5 | Laurel | May 17 2018 | 43°28'49.40"N | 80°36'15.37"W |
| 6 | Claire Lake Outflow | May 25, 2018 | 43°27'47.27"N | 80°33'7.83"W |
