# Supplementary material for: COI metabarcoding primer choice affects richness and recovery of indicator taxa in freshwater systems
Source: PLoS One. 2019 Sep 12;14(9):e0220953. doi: 10.1371/journal.pone.0220953 (PMC6742397; doi:10.1371/journal.pone.0220953)
Supplement: S2 Table — (DOCX) [file pone.0220953.s002.docx]

**Table S2: Reads counts for all taxa**

|  | BR5 | F230R | ml-jg | BF1 | BF2 | fwh1 | Total |
| --- | --- | --- | --- | --- | --- | --- | --- |
| Raw | N/A | N/A | N/A | N/A | N/A | N/A | 9,980,584 x 2 |
| Paired | N/A | N/A | N/A | N/A | N/A | N/A | 8,253,974 |
| Primer trimmed | 1,113,306 | 1,523,753 | 1,492,705 | 1,774,254 | 941,889 | 773,201 | 7,619,108 |

Raw – Reads off the sequencer

Paired – After merging forward and reverse reads

Primer-trimmed – After removing primers and a mimimum paired length of 150 bp

N/A – Not applicable, as the markers were pooled before sequencing, then sorted by primer sequence at the primer trimming step
