# Supplementary material for: COI metabarcoding primer choice affects richness and recovery of indicator taxa in freshwater systems
Source: PLoS One. 2019 Sep 12;14(9):e0220953. doi: 10.1371/journal.pone.0220953 (PMC6742397; doi:10.1371/journal.pone.0220953)
Supplement: S3 Table — (DOCX) [file pone.0220953.s003.docx]

**Table S3: ESV counts for all taxa**

|  | BR5 | F230R | ml-jg | BF1R2 | BF2R2 | fwh1 | Total |
| --- | --- | --- | --- | --- | --- | --- | --- |
| ESVs | 3,494 | 2,605 | 3,305 | 6,139 | 3,491 | 1,944 | 20,978 |
| Reads in ESVs | 324,721 | 643,405 | 375,119 | 432,848 | 80,886 | 457,968 | 2,314,947 |
| Proportion of raw reads (%) | 3.3 | 6.4 | 3.8 | 4.3 | 0.8 | 4.6 | 23.2* |

* ~ 77% of raw reads removed during denoising (putative sequence errors, chimeras, PhiX contamination, rare singletons and doubletons
