# Supplementary material for: COI metabarcoding primer choice affects richness and recovery of indicator taxa in freshwater systems
Source: PLoS One. 2019 Sep 12;14(9):e0220953. doi: 10.1371/journal.pone.0220953 (PMC6742397; doi:10.1371/journal.pone.0220953)
Supplement: S4 Table — (DOCX) [file pone.0220953.s004.docx]

**Table S4: Filtering for high confidence Arthropoda identifications affects the proportion of assignments retained across taxonomic ranks**

|  | ESVs | Species | Genus | Family | Order | Class |
| --- | --- | --- | --- | --- | --- | --- |
| High confidence assignments* | 4,940 | 120 | 100 | 69 | 44 | 9 |
| All unique assignments | 4,940 | 1049 | 660 | 270 | 44 | 9 |
| Proportion of assignments retained after applying bootstrap support cutoffs (%) | 100 | 11.4 | 15.2 | 25.6 | 100 | 100 |
| Reads in high confidence assignments | 1,280,397 | 718,120 | 775,884 | 1,012,057 | 1,280,397 | 1,280,397 |
| Proportion raw reads in high confidence assignments (%) | 12.8 | 7.2 | 7.8 | 10.1 | 12.8 | 12.8 |

* ESVs not taxonomically assigned so no bootstrap support filtering required; Species >= 0.70 bootstrap support cutoff; Genus >= 0.30; Family >=0.20; No bootstrap support filtering needed at the order or class ranks to ensure 99% correct assignments (95% for species)
