# Supplementary figures and images for: COI metabarcoding primer choice affects richness and recovery of indicator taxa in freshwater systems
Source: PLoS One. 2019 Sep 12;14(9):e0220953. doi: 10.1371/journal.pone.0220953 (PMC6742397; doi:10.1371/journal.pone.0220953)

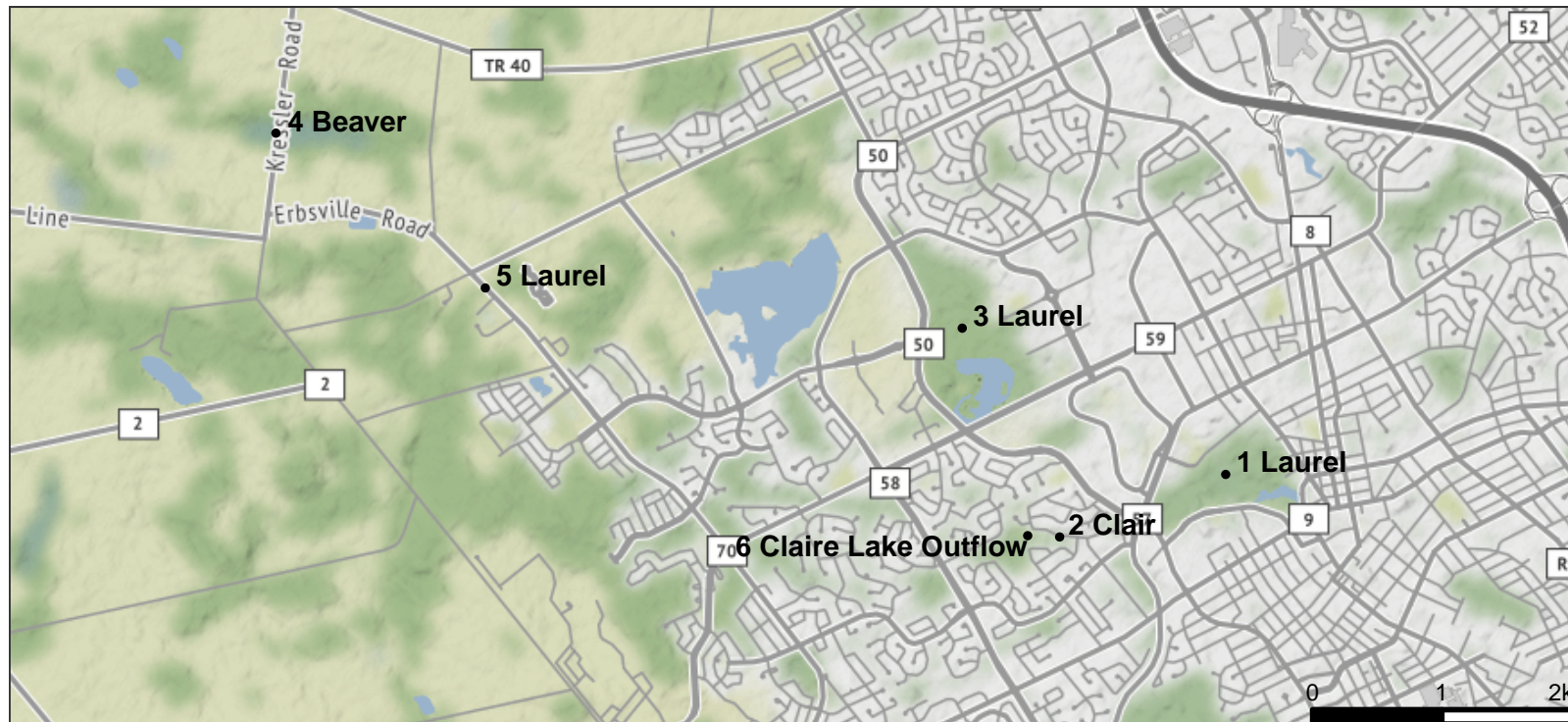

Supplement: S1 Fig — The map shows where collection sites were located. We used the ggmap library in R with the ‘get_stamenmap’ function to create the map (Kahle and Wickham, 2013 The R Journal 5 (1): 144–161), then used the ggsn library to add a scale bar (Santos Baquero, 2019 https://CRAN.R-project.org/package=ggsn). (PDF) [file pone.0220953.s005.pdf]

## A) All phyla

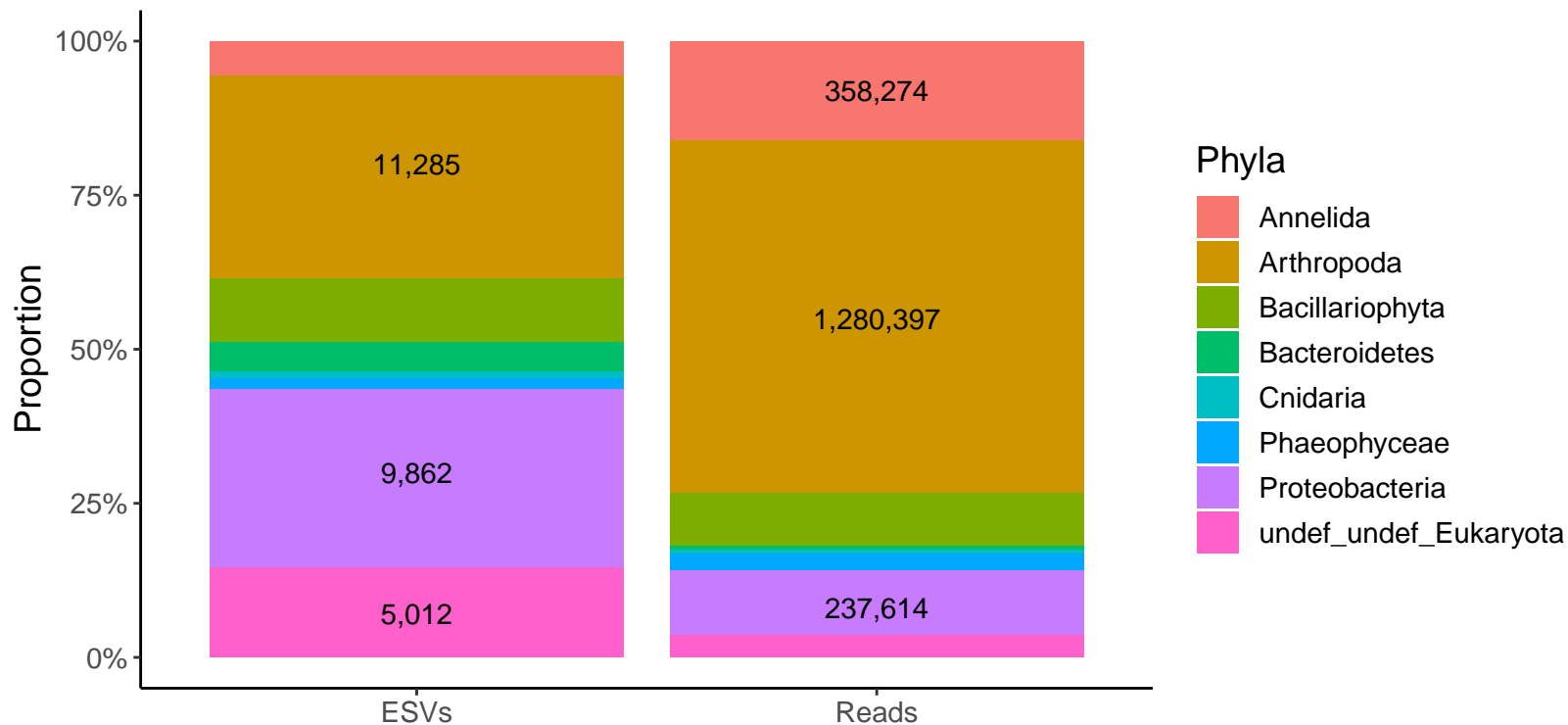

## B) Arthropoda taxa

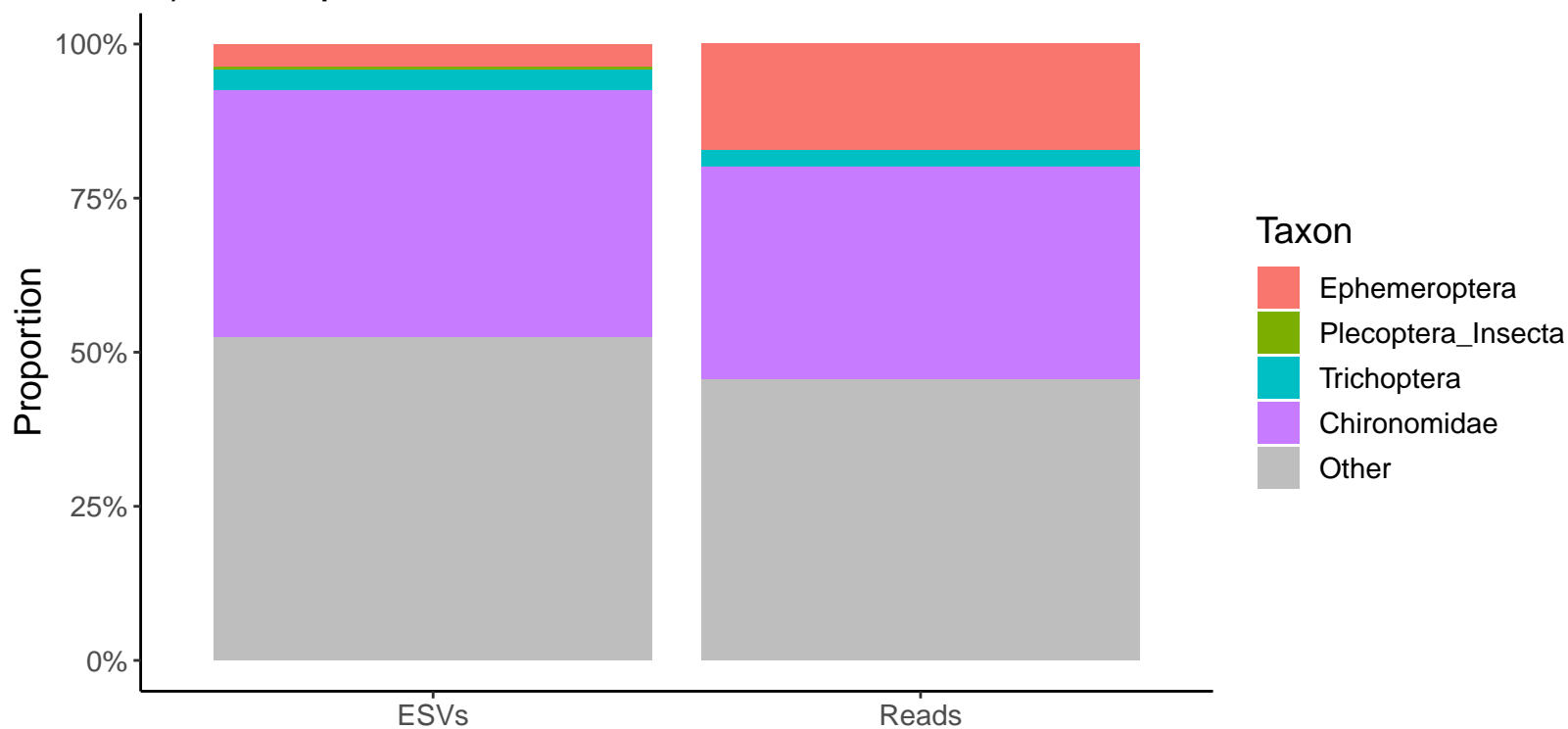

Supplement: S2 Fig — Number of ESVs and reads in ESVs are shown for: a) all taxa at the phylum rank, and b) Arthropoda focusing on the Ephemeroptera, Plectoptera, Trichoptera, and Chironomidae. Results summarize the denoised ESVs across all samples, before rarefaction. (PDF) [file pone.0220953.s006.pdf]

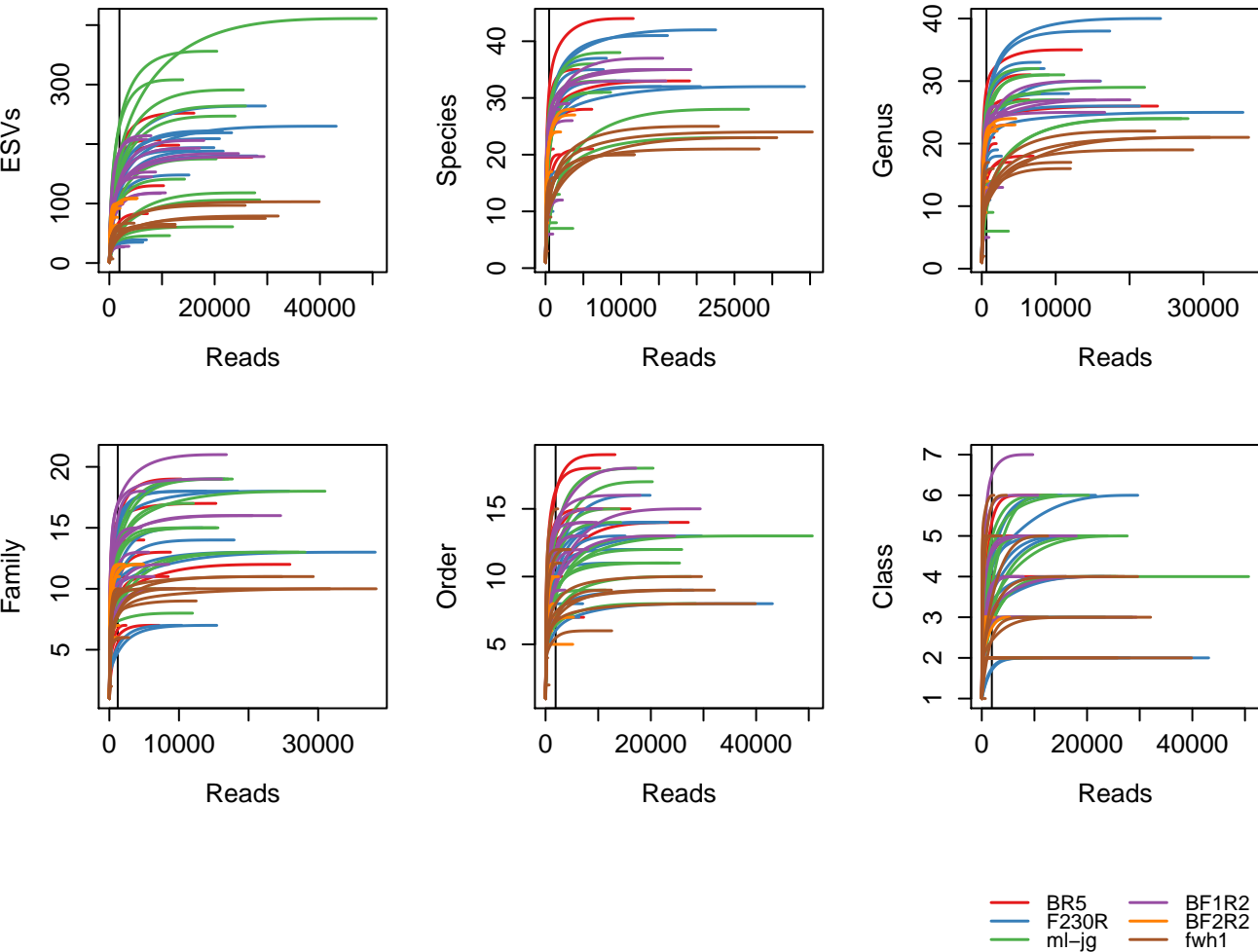

Supplement: S3 Fig — For each primer, color coded as in the legend, there are 12 lines for the six field sampling sites x 2 PCR replicates. The vertical line indicates the 15th percentile library size that was used to normalize variable library sizes for subsequent diversity analyses. ESVs = exact sequence variants. (PDF) [file pone.0220953.s007.pdf]

ESV Richness

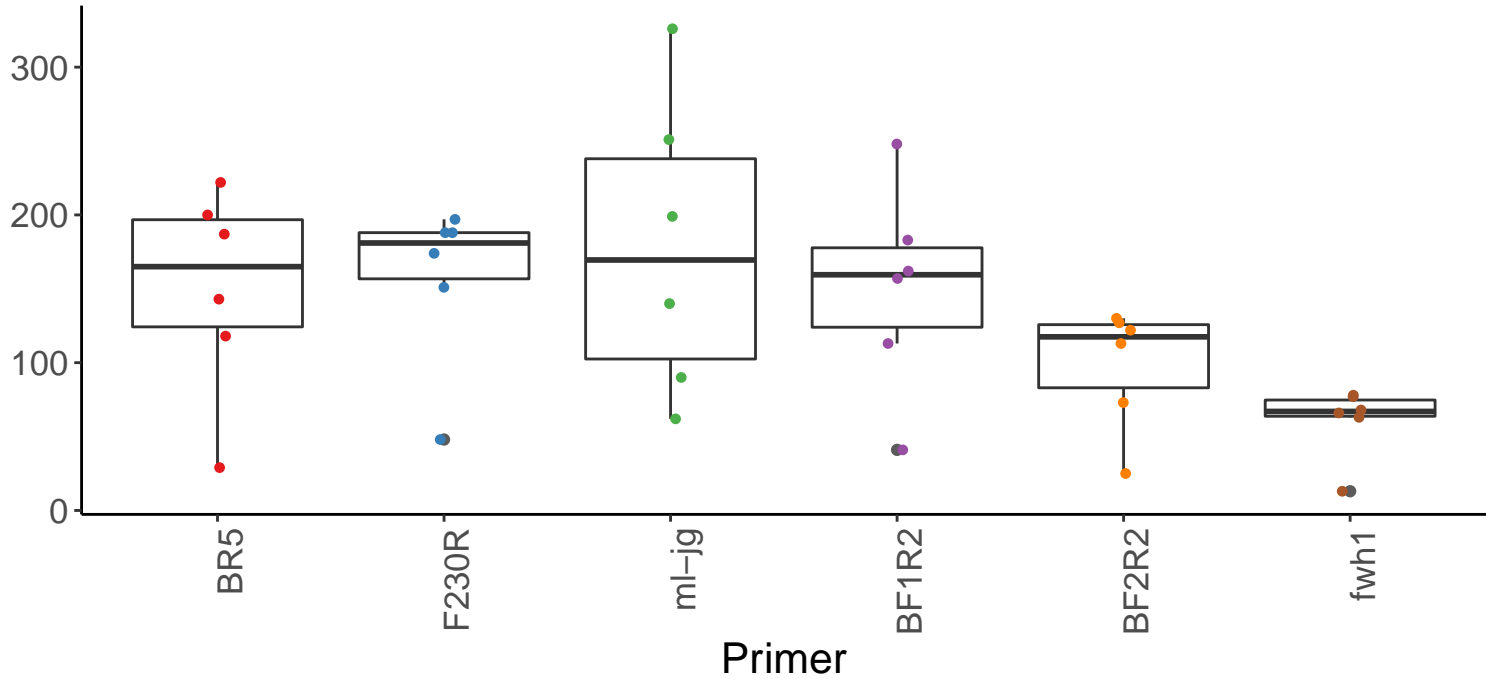

Supplement: S4 Fig — The first panel shows the six COI markers tested. Based on normalized data. Results shown are for 2 pooled PCR replicates at the ESV rank. ESV = exact sequence variant. (PDF) [file pone.0220953.s008.pdf]

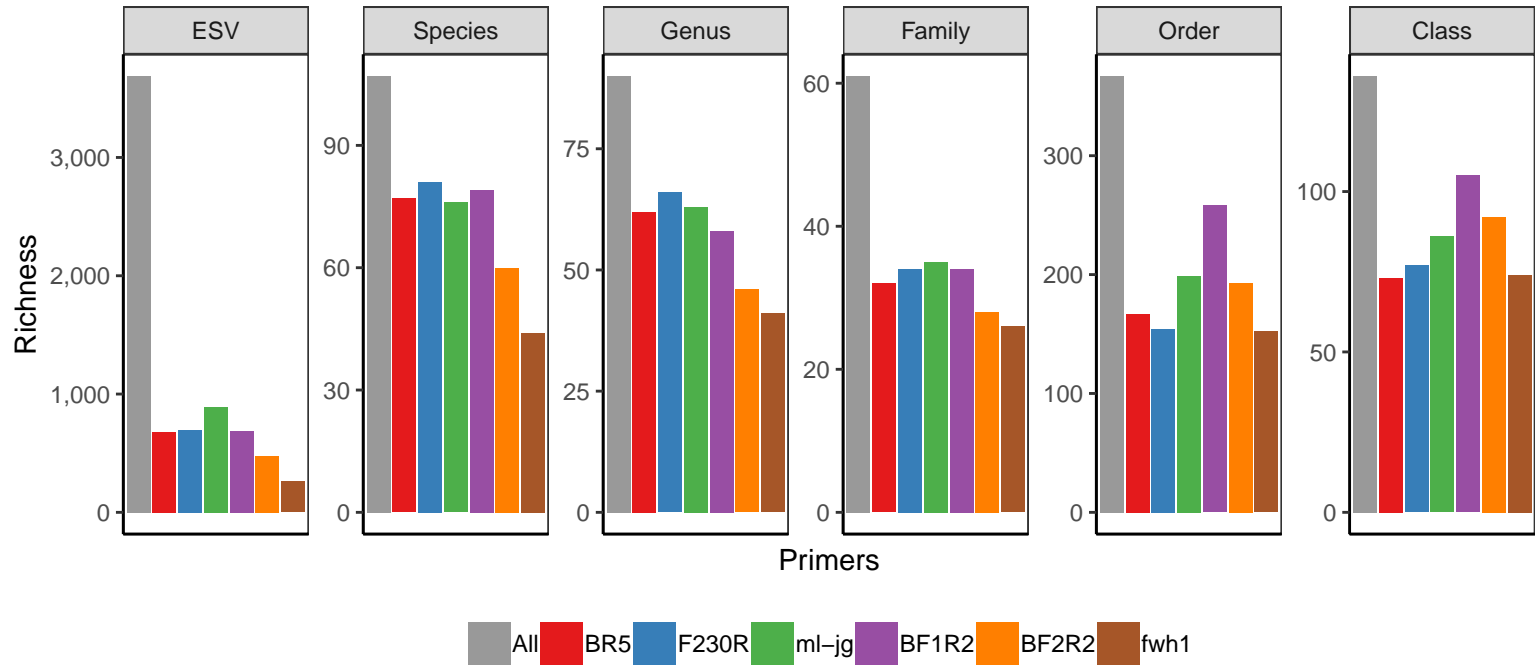

Supplement: S5 Fig — Richness from each COI marker at a variety of taxonomic ranks are shown. Results are based on normalized data. ESV = exact sequence variant. (PDF) [file pone.0220953.s009.pdf]

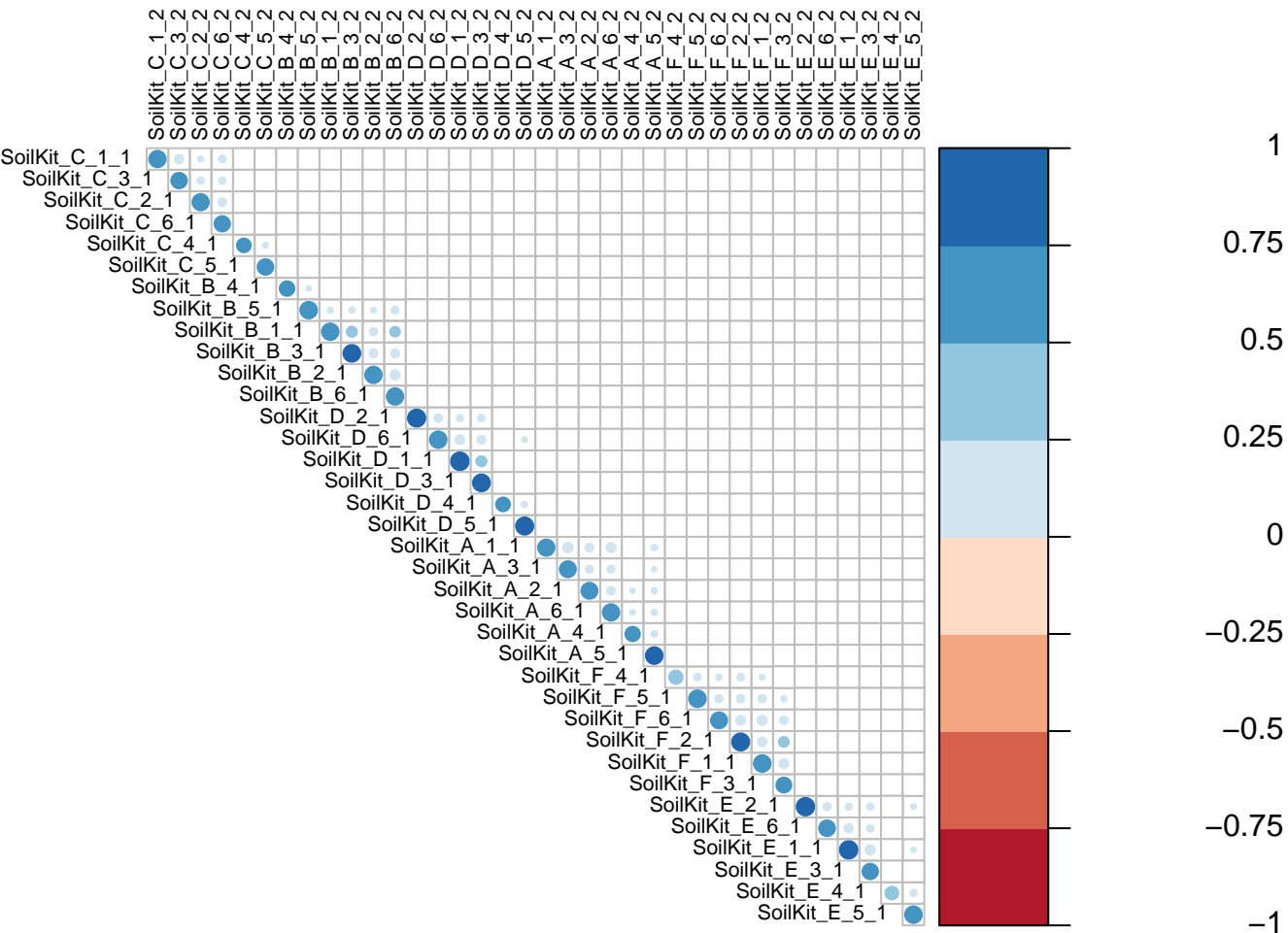

Supplement: S6 Fig — Circle color and size reflect Pearson correlations. Only significant correlations with a p-value < = 0.05 are shown. Results are based on normalized data. ESV = exact sequence variants. Label naming convention is as follows: DNA extraction kit _ marker _ site _ PCR replicate. A = BR5, B = F230R, C = ml-jg, D = BF1, E = BF2, F = fwh1. (PDF) [file pone.0220953.s010.pdf]
